# Supplementary material for: Autophagy Deficiency in Renal Proximal Tubular Cells Leads to an Increase in Cellular Injury and Apoptosis under Normal Fed Conditions
Source: Int J Mol Sci. 2019 Dec 25;21(1):155. doi: 10.3390/ijms21010155 (PMC6982095; doi:10.3390/ijms21010155)
Supplement: Supplementary file 1 [file ijms-21-00155-s001.pdf]

Supplementary Materials:

Autophagy Deficiency in Renal Proximal Tubular Cells Leads to an Increase in Cellular Injury and Apoptosis under Normal Fed Conditions

Chigure Suzuki [1,2], Isei Tanida [1,*], Juan Alejandro Oliva Trejo [1], Soichiro Kakuta [1,3] and Yasuo Uchiyama [1,*]

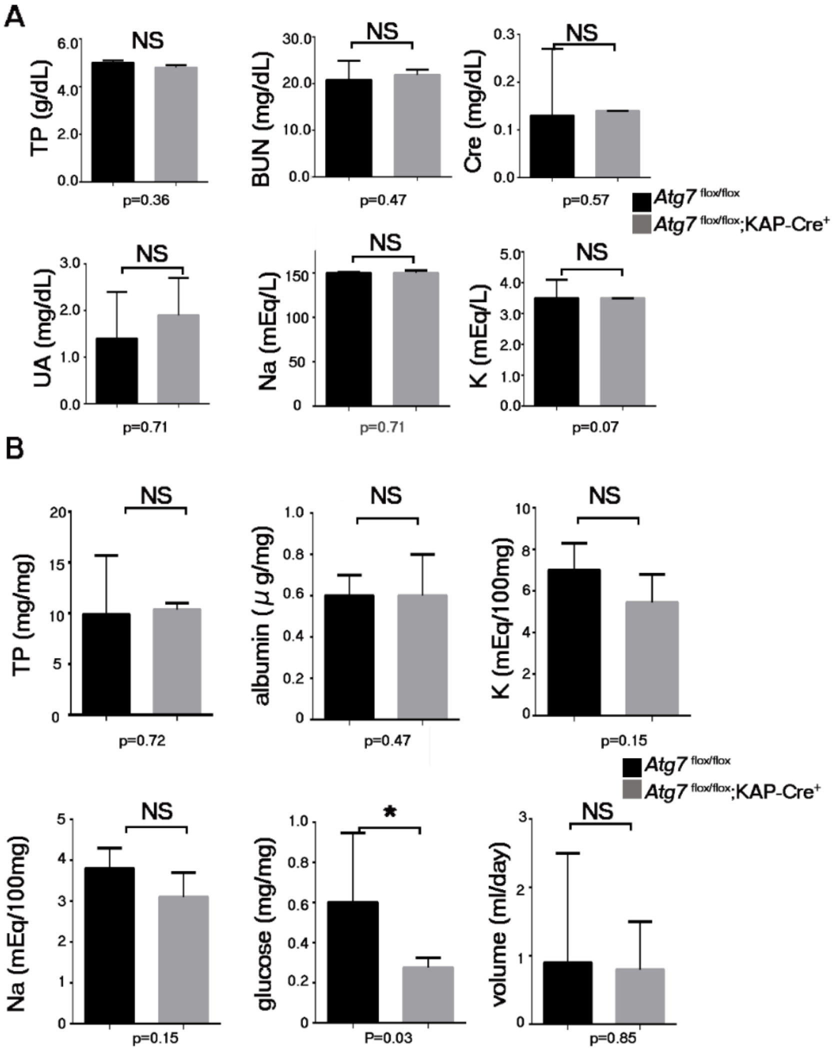

Figure S1. ***Atg7$^{flox/flox}$;KAP-Cre^{+}* mice had no overt abnormality in the blood and urine exams for renal function. (A)** Blood analysis data of 6 months old *Atg7*$^{flox/flox}$ and *Atg7*$^{flox/flox}$*;KAP-Cre*$^{+}$ mice. **TP**: Total protein, **BUN**: urea nitrogen, **Cre**: creatinine, **UA**: uric acid, **Na**: sodium, **K**: potassium. $N = 3$ to 4 mice. (**B**) 24 hours urinary data of Total Protein (**TP**), **albumin**, potassium (**K**), sodium (**Na**) **glucose**, and **urine volume**. TP, albumin, K, Na describe as ratio of urinary

creatinine. N = 4 mice. Data in graphs are expressed as the median ± SEM. Statistical analyses were performed using Statistical analyses were performed using a python software with scipy. stats module. Data in the graph are expressed as the median ± RANGE, and statistical significance was set at $p < 0.05$. **NS**: no significant difference.

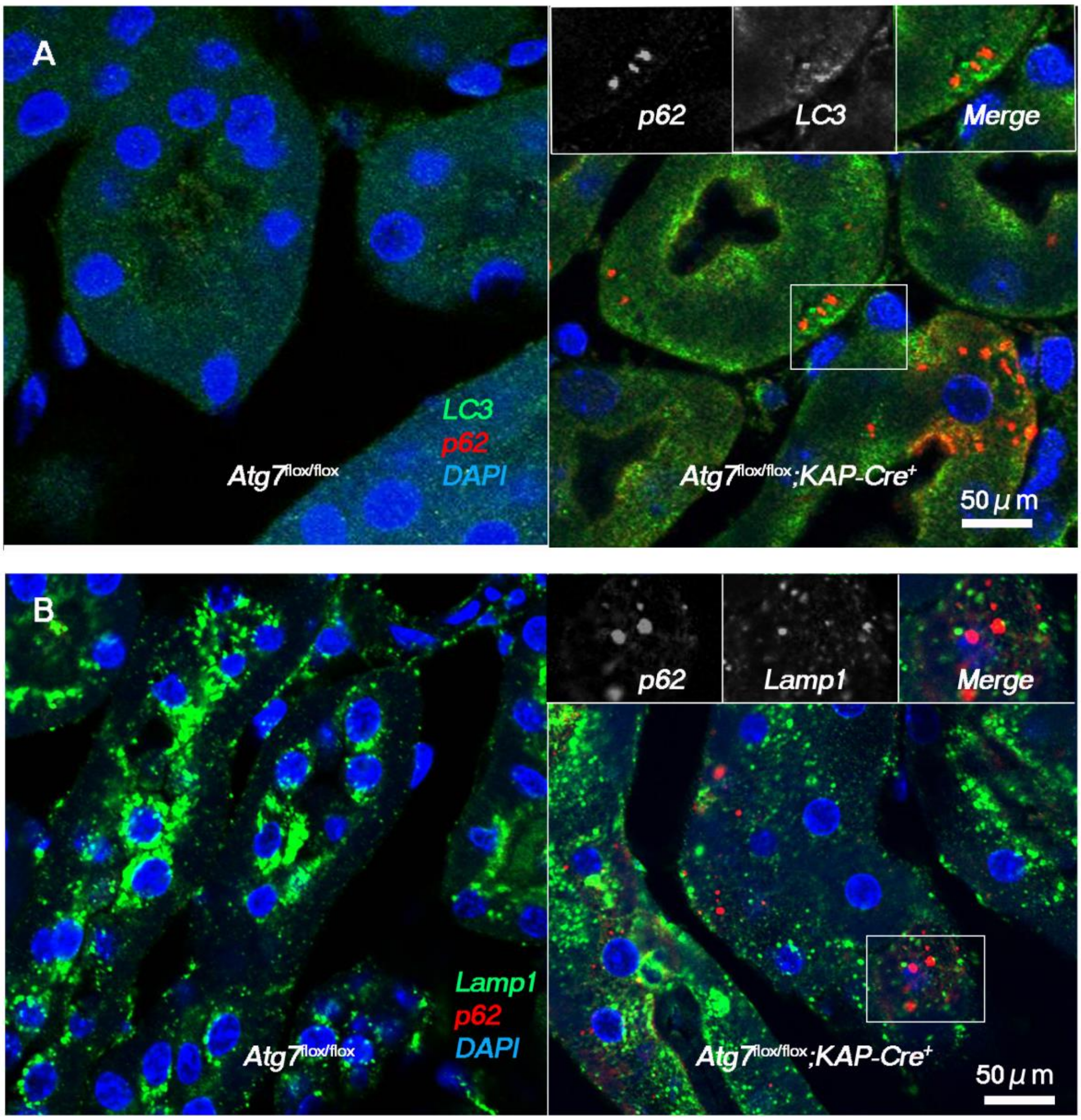

Figure S2. Autophagy flux was impaired in renal tubular cells of ***Atg7***$^{\text{flox/flox}}$***;KAP-Cre***$^{+}$ **mice.** Representative images of the proximal region of the kidney of ***Atg7***$^{\text{flox/flox}}$***;KAP-Cre***$^{+}$ **mice (**left panels**) and** ***Atg7***$^{\text{flox/flox}}$ **mice (**right panels**)** stained with: (**A)** **LC3** (green) and **p62** (red). Nuclei were stained with **DAPI (**blue). (**B) Lamp1** (green) and **p62** (red). Nuclei were stained with **DAPI** (blue).

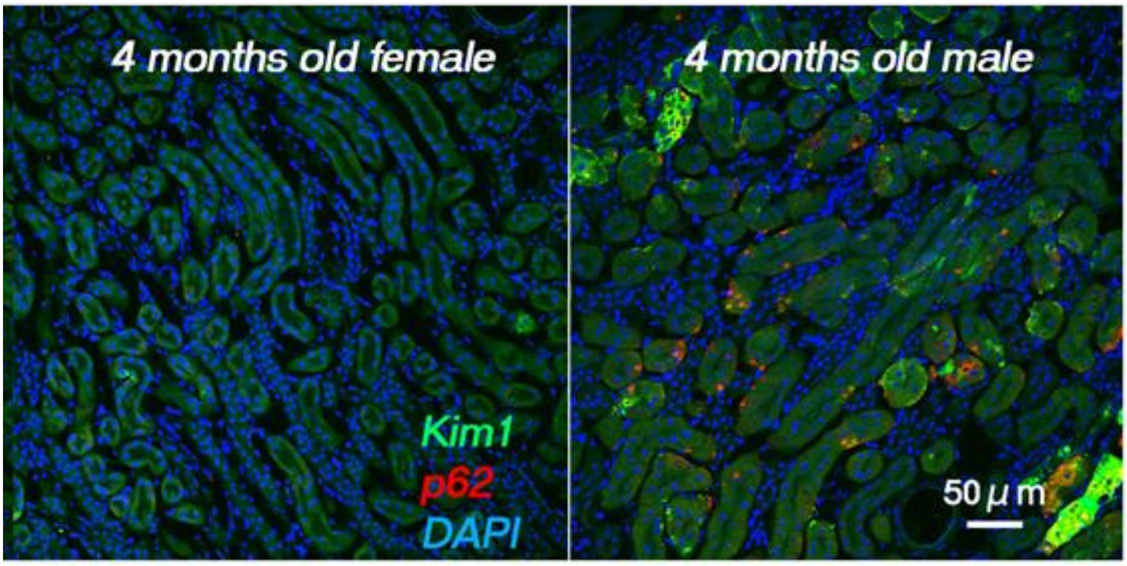

Figure S3. Female ***Atg7*$^{flox/flox}$*;KAP-Cre*$^{+}$ mice had no accumulation of p62 and increment of Kim1 positive immunosignals in their proximal tubular cells.** Representative images of kidney samples of 4 months old **female** (left) and **male** (right) ***Atg7*$^{flox/flox}$*;KAP-Cre*$^{+}$** mice stained with **Kim-1** (green) and **p62** (red). Nuclei were stained with **DAPI** (blue).
